# Post-graduation Plans of Undergraduate BME Students: Gender, Self-efficacy, Value, and Identity Beliefs

**DOI:** 10.1007/s10439-020-02693-9

**Published:** 2020-11-23

**Authors:** Anita Patrick, Maura Borrego, Catherine Riegle-Crumb

**Affiliations:** 1 The Center for Engineering Education, The University of Texas at Austin, 2501 Speedway, Austin, TX 78712 USA; 2 Department of Curriculum and Instruction, STEM Education, The University of Texas at Austin, Austin, TX USA

**Keywords:** Women, Biomedical engineering education, Bioengineering education, Cluster analysis, Survey, Career choice, Motivation

## Abstract

This study investigates career intentions and students’ engineering attitudes in BME, with a focus on gender differences. Data from *n* = 716 undergraduate biomedical engineering students at a large public research institution in the United States were analyzed using hierarchical agglomerative cluster analysis. Results revealed five clusters of intended post-graduation plans: *Engineering Job and Graduate School, Any Job, Non-Engineering Job and Graduate School, Any Option,* and *Any Graduate School.* Women were evenly distributed across clusters; there was no evidence of gendered career preferences. The main findings in regard to engineering attitudes reveal significant differences by cluster in interest, attainment value, utility value, and professional identity, but not in academic self-efficacy. Yet, within clusters the only gender differences were women’s lower engineering academic self-efficacy, interest and professional identity compared to men. Implications and areas of future research are discussed.

## Introduction

Recent national reports continue to document how engineering needs to attract the best and brightest students to enhance economic productivity and improve quality of life worldwide.^11^ Arguments for diversity in engineering range from the competitive advantages of having more critical thinkers, inventors, and problem-solvers in the field, to promoting social justice and equity.^2^ Having a more diverse population of engineers may help encourage more women and minorities to pursue this area of study and eventually join the engineering workforce. The practical implications are that once a critical mass of those who are traditionally under-represented in engineering enter and remain in the engineering workforce, this will then lead to more innovative solutions to the grand challenges facing society.^2^ Yet despite this acknowledged need for diversity, engineering still has one of the lowest percentages of women degree holders among STEM fields^24^ and women account for only 13% of the total U.S. engineering workforce.^6^

Yet, within engineering, Bio/biomedical engineering (BME) stands out as a field that has attracted many women, at both undergraduate and graduate levels. As seen in Table 1, in 2018, of all engineering bachelor’s degrees awarded in the U.S., only 21.9% were awarded to women.^32^ Similarly, women earned 26.7% of master’s degrees and 23.6% of doctoral degrees in engineering. However, BME awarded 45.4, 44.1 and 39.7% of degrees to women in these respective categories. With regard to bio/biomedical engineers’ impact on the workforce, the U.S. Bureau of Labor Statistics reported a total of 19,800 jobs in Biomedical Engineering in 2018 with a projected growth of 4%, approximately 700 jobs, from 2018 to 2028.^7^ Further, BME graduates go on to pursue a variety of career paths in and outside of engineering. Biomedical engineers are found in health and medical-related occupations, which include working as scientists developing medical therapeutics including artificial organs; medical researchers designing instruments, devices, and software for healthcare; technicians installing and maintaining biomedical equipment, and sales managers in industry manufacturing.^7^

**Table 1:** Percentage of degrees awarded in engineering and bio/biomedical engineering (BME) by gender. *Source* Roy (2018). Engineering by the numbers. American Society for Engineering Education.

Degree by gender	All engineering majors^+^	BME majors^++^	BME of all engineering majors
Bachelor’s			
Men	78.1	54.6	2.8
Women	21.9	45.4	2.4
All	100.0	100.0	5.2
Master’s			
Men	73.5	55.9	2.2
Women	26.5	44.1	1.7
All	100.0	100.0	3.9
Doctoral			
Men	76.2	60.3	5.1
Women	23.8	39.7	3.3
All	100.0	100.0	8.4

^+^The number of degrees awarded are as follows: Bachelor’s (136,233), Master’s (66,340), and Doctoral (12,156)

^++^The number of degrees awarded are as follows: Bachelor’s (7130), Master’s (2568), and Doctoral (1025)

Yet, tracking biomedical engineering employment into the engineering workforce is less precise as there are shared job titles that may or may not include “engineer” or “engineering” in the title. Further, while the field is roughly gender equitable in terms of bachelor degree attainment, the exact data on where women go within these occupational areas is unknown.^18^ While research on engineering more broadly finds that female engineers are less likely than male engineers to be in engineering occupations,^24^ it is not evident whether patterns for BME follow this larger trend, or whether instead, given the varied potential careers that BME graduates can and do pursue, whether men and women in BME may be similarly distributed across different post-graduate career options. As such, BME is an opportune discipline to study potential gender differences in intended career plans in a more nuanced, non-binary way that considers the multiple career paths that students might simultaneously consider, rather than simply whether individuals stay or leave engineering. Further, this study makes a new contribution to research in this area by examining whether and how students’ various intended career plans are related to their engineering attitudes, and whether there are gender differences in attitudes even among those with similar career plans.

### Theoretical Framework

We argue that the way that prior research typically conceptualizes engineering career choice as a binary decision to either stay or leave is an oversimplification.^8^ It has been documented that BME graduates go on to pursue both engineering, engineering-related and non-engineering career paths.^17^,^31^ A better characterization of students’ intended post-graduation plans would describe students’ decisions without privileging engineering as the most desirable choice compared to other options. This view helps to operationalize more nuanced categorizations of career choice in engineering beyond persisters and non-persisters.^20^,^34^

To inform our examination of potential gender differences in BME career plans, there are a number of theories of career choice and persistence in STEM. Our choice of constructs (Table 2) is guided by those found in prior research; we draw primarily from expectancy-value theory (EVT)^13^ and research on engineering identity to guide our focus on attitudes (including interest, attainment value, utility value, and academic self-efficacy), and professional identity as they relate to engineering.

**Table 2:** Descriptors of key terms in this study.

Term	Literature	Definition
Post-graduation plans	Achievement-related choice and persistence (EVT)	Relating to future career paths following the completion of an undergraduate engineering degree
Engineering interest	Intrinsic value (EVT); Interest (identity)	Wanting to know or learn more about engineering
Engineering attainment value	Attainment task value (EVT)	How much a task is important to current and future goals in engineering
Engineering utility value	Utility task value (EVT)	How useful a task is to current and future goals in engineering
Engineering academic self-efficacy	Self-concept of one’s abilities (EVT); performance/ competence (identity)	Confidence in one’s abilities to complete a particular task or be successful in a given situation in engineering
Engineering professional identity (EPI)	Self-Schemata (EVT); Engineering identity (identity)	Defining the overlap between an individual’s personal identity and the identity of an engineer

EVT abbreviates expectancy-value theory

#### Subjective Task Values: Interest, Utility and Attainment Value

Of the factors that affect departure from STEM disciplines, loss in interest—particularly among females–has been cited as a primary reason.^33^ Interest (or lack thereof) has implications at the undergraduate level even among those already self-selected into engineering majors. For example, in a longitudinal study, self-reported interest in engineering was positively associated with an increased likelihood of 1-year persistence (as defined by continued enrollment in the major) in engineering.^26^ Engineering interest was also found to be a significant predictor of intending to pursue engineering careers post-graduation for senior engineering majors.^34^ Additionally, attainment value (how important it is to pursue a given domain) and utility value (how useful a domain is perceived to be) are other subjective task values that are components of EVT, and are frequently studied together. For instance, in a qualitative study of undergraduate engineering students, attainment value, utility value, and interest were all associated with changes in students’ persistence over a 4-year period. Moreover, the authors found different gendered combinations of motivating beliefs that governed students’ choice to persist in an engineering major.^22^

#### Academic Self-efficacy

Extensive research has been conducted on the role that self-efficacy plays in gender differences in educational choices and persistence in STEM.^10^ Consistently, research shows that women have lower academic self-efficacy than men.^39^ Further, even among women already self-selected into engineering, self-efficacy continues to play an important role in academic outcomes. For example, while women’s self-reported intentions to persist in engineering were positively and significantly related to multiple measures of engineering self-efficacy, gender gaps in self-efficacy also contributed to gender differences in intentions.^21^ Additionally, students’ academic self-efficacy beliefs have been linked to a return to engineering graduate school five years after students completed an undergraduate degree.^27^

#### Engineering Identity

Engineering identity, like other STEM identities, has been studied to investigate its role on outcomes such as career choice. For example, research finds that engineering identification, defined as the extent to which students define themselves through their role or performance in engineering, is a significant predictor of intention to persist in an engineering career.^15^ Specific to BME, the study of engineering identity has been ongoing over the last several years with particular focus on how BME students negotiate their engineering identity with other identities such as clinician^14^ and scientist.^35^ While prior studies have not yet linked gender differences in identity to specific career choices, men are consistently found to have higher levels of engineering identity than women.^23^

### Research Questions

In the present study, we examine undergraduate biomedical engineering students to examine their career intentions. This study addressed the following research questions:

1. Do the intended post-graduation career plans of undergraduate biomedical engineering students cluster into groupings that clearly differentiate those who intend to stay in engineering vs. those who intend to leave engineering, or is there evidence that students form clusters based on more complex future plans?
2. If career plans do differentiate students into clusters, is there evidence that cluster membership varies according to student gender, as well as students’ engineering attitudes (interest, attainment value, utility value, academic self-efficacy, and professional identity)?
3. Are there gender differences in these engineering attitudes *within* each cluster?

## Materials and Methods

### BME Department Characteristics

The setting was a large public institution in the U.S. with high-ranking engineering programs where the students are admitted directly into specific majors (there is no general or first-year engineering program). The BME department under study was established in 2001. As example of the program size in a given year, the total undergraduate enrollment in 2017 was 555 students of which 54% were male and 46% were female students. Based on information collected each year from graduating seniors, the department historically has students who pursue a variety of career plans including further education in graduate or professional school and industry jobs in government or the private sector. This pattern is not atypical for BME programs, since BME programs have produced a heterogeneity of practitioners both within and beyond engineering.^1^

### Data Collection

#### Participants

This study was conducted under IRB approval. To accumulate a larger dataset, survey responses were collected during the first month of class in three consecutive fall semesters: 2016, 2017, and 2018. Students were administered a survey, which took approximately fifteen minutes to complete, in class *via* Qualtrics. Based on university student identification numbers collected *via* the survey, we removed duplicate responses by retaining the earliest response for students who completed multiple surveys. Only students who consented were included as part of the analytical sample (Table 3). This resulted in 716 unique students in the dataset.

**Table 3:** Student background variables by gender (reported in percentages).

Variable	Men *n* = 373	Women *n* = 343	Total *n* = 716
Gender	52.1	47.9	100
Student classification^+^			
Lower division	33.7	30.6	64.3
Upper division	18.4	17.3	35.7
Race			
White	23.9	22.8	46.7
Asian	20.7	17.7	38.4
Hispanic	5.2	5.3	10.5
Multiracial	1.5	1.1	2.6
Black	0.7	1.0	1.7
American Indian/Alaskan Native	0.1	0.0	0.1
Native Hawaiian/Pacific Islander	0.0	0.0	0.0

^+^Lower division (1st and 2nd year); upper division (3rd and 4th year)

#### Student Characteristics

Demographic data was gathered from self-report. Gender was coded dichotomously: “0” for male students and “1” female students. Student classification was coded dichotomously: “0” for lower division students and “1” upper division students. Division is based on students’ course level (lower: 1st and 2nd year; upper: 3rd and 4th year). Addressing student classification has been used elsewhere,^34^ and research suggests that even late into their senior year students are unsure of their post-graduation plans.^16^ Race/ethnicity was dummy-coded for each self-reported category listed in the survey. All racial/ethnic categories were mutually exclusive except Multiracial which was defined as participants reporting two or more non-Hispanic categories.

### Variables

Analyses include non-duplicate responses with complete data on gender, engineering professional identity, engineering academic self-efficacy, engineering interest, engineering attainment value, engineering utility value, and the post-graduation plan variables. Each of the variables explored are measured as self-reported by students since they represent attitudes rather than skills or knowledge.

#### Intended Post-graduation Plan Variables

To address the research questions, we used the same four categories as Sheppard *et al.*^34^ Students were asked “how likely it is that you would do each of the following after graduation?” (a) “work in an engineering job”, (b) “work in a non-engineering job”, (c) “go to graduate school in an engineering discipline”, and (d) “go to graduate school outside of engineering”. The scale was from 1 (definitely not) to 5 (definitely yes). Students rated each option individually. These four items were the input variables by which cluster group membership was determined.

#### Engineering Attitude Variables

For the list of the items in each scale and the Cronbach alpha reliability (see Table 4). Engineering academic self-efficacy and engineering interest were taken with slight modifications from a previous study of engineering identity, which used items typically used to measure math or science attitudes and adapted them for engineering.^26^ Both scales were measured on a 5-point Likert scale from 1 (strongly disagree) to 5 (strongly agree). Engineering attainment and utility value were taken from a subscale from work with expectancy-value theory,^38^ adapted by replacing “math” with “engineering.” These items were measured on a 5-point scale from 1 (not at all) to 5 (very). Engineering professional identity (EPI) was a variable composed of two questions (measured on a scale of 1 to 8) from previously validated items.^5^

**Table 4:** List of engineering attitude variables.

Factor	Item	Alpha reliability
Engineering academic self-efficacy	I can understand concepts I have studied in engineering	0.88
	I am confident that I can understand engineering in class	
	I can overcome setbacks in engineering	
	I am confident that I can understand engineering outside of class	
	I can do well on exams in engineering	
Engineering interest	I enjoy learning engineering	0.75
	I am interested in learning more about engineering	
Engineering attainment value	Compared to other activities, how important is it for you to be good at engineering?	0.80
	For me, being good in engineering is important.	
Engineering utility value^+^	In general, how useful is what you learn in engineering?	–
Engineering professional identity	1)Please describe your relationship with engineering by using the following diagrams. Imagine that the circles at the left represent your own personal identity (i.e., what describes you as a unique individual), while the circles at the right represent the identity of an engineer (i.e., what describes an engineer). Which diagram best describes the level of overlap between your own identity and the identity of an engineer?	0.82
	To what extent does your own sense of who you are (i.e., your personal identity) overlap with your sense of what an engineer is (i.e., the identity of an engineer)? [1 “not at all” to 8 “to a great extent”]	

^+^This was a single-item variable. Alpha reliability cannot be calculated for single-item variables

### Data Analysis

To answer research question 1, we conducted a multivariate classification procedure known as cluster analysis. By definition, classification refers to the division of a larger heterogeneous group into smaller, homogenous groups where members are similar to each other while different from the cases in the other groups.^12^

We specifically conducted hierarchical agglomerative cluster analysis (HACA) with complete-linkage clustering. HACA utilizes a bottom-up methodology that adds one observation at a time into the algorithm until all observations are merged into a single cluster while at the same time determining which values are most dissimilar from one another *via* the linkage algorithm.^19^ HACA with complete-linkage clustering produces compact clusters, characterized by how dissimilar one cluster is from another. The advantage of this technique is that the researcher gets a qualitative “feel” of the data by visually identifying breaks in the graph called a dendrogram (i.e., a hierarchical cluster tree diagram), denoting different groups. Simply, hierarchical cluster analysis allows for a more intuitive determination of clusters when there is not a presupposed number of clusters expected in the data.

The procedure for this analysis is broadly conducted in three parts. First, we conducted complete-linkage clustering algorithm to produce a hierarchical cluster tree that shows the relationship of the input data based on students’ rating on each of four intended post-graduation plans (work in an engineering job, work in a non-engineering job, go to graduate school in engineering, and go to graduate school outside of engineering). Next, we visually determined the appropriate cut value that split the tree (dendrogram) into *k* clusters. Last, we confirmed *k* clusters by examining the cluster stop rules for hierarchical cluster analysis: Calinski-Harabasz pseudo-F and Duda-Hart pseudo-*T*-squared. Distinct clusters are characterized by large Calinski–Harabasz pseudo-*F* values, large Duda–Hart Je(2)/Je(1) values, and small Duda–Hart pseudo-*T*-squared values.^36^ Once the clusters were identified we characteristically named each one.

To answer research question 2, we then examined differences in gender representation within and between these defined clusters using t-tests and ANOVAs. Lastly, we conducted ANOVAs with post-hoc t-tests on engineering attainment value, engineering utility value, engineering professional identity, engineering interest, and engineering academic self-efficacy to determine if there were significant differences in the mean values of each variable between clusters. To answer research question 3, we conducted t-tests on these five engineering attitudes by gender to determine if there are significant differences in the mean values of each attitude within clusters. We also reported effect sizes.

## Results

### Cluster Analysis Results

**Figure 1:**
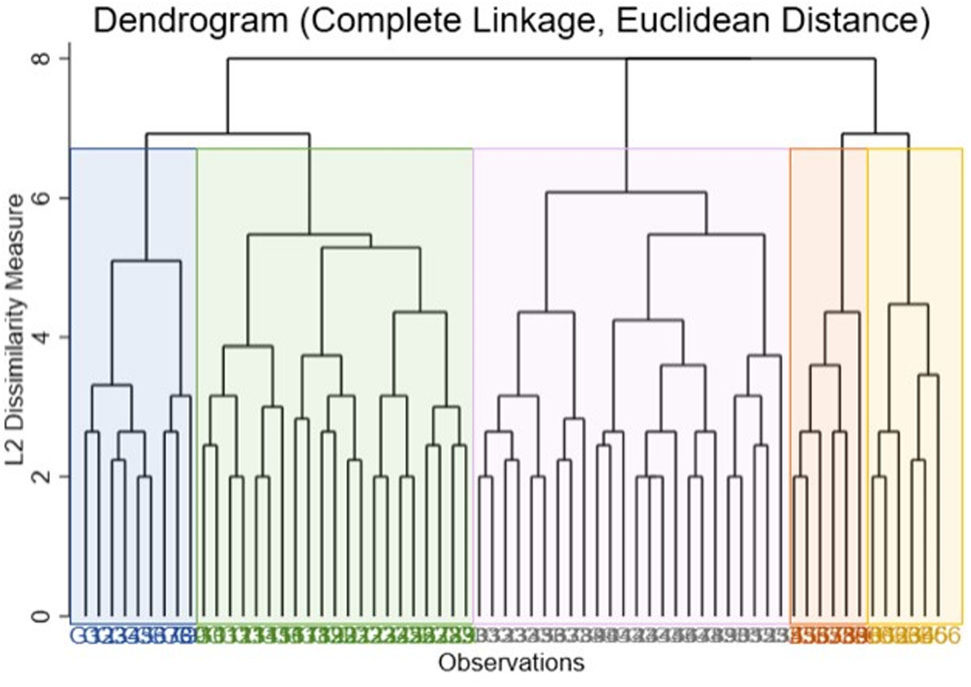
Dendrogram of BME students’ intended post-graduations plans. Colored blocking is superimposed on the dendrogram to visually segment and illustrate the branches of the 5-cluster solution.

Upon examination of the dendrogram (Fig. 1), we interpreted at minimum a 3-cluster solution. However, quantitatively, 5 clusters were found to be the best solution as evidenced by values of the Calinksi/Harabasz (pseudo-*F* = 169.12) and Duda/Hart (Je(2)/Je(1) = 81.50; pseudo *T*-squared = 73.08) statistics.

**Figure 2:**
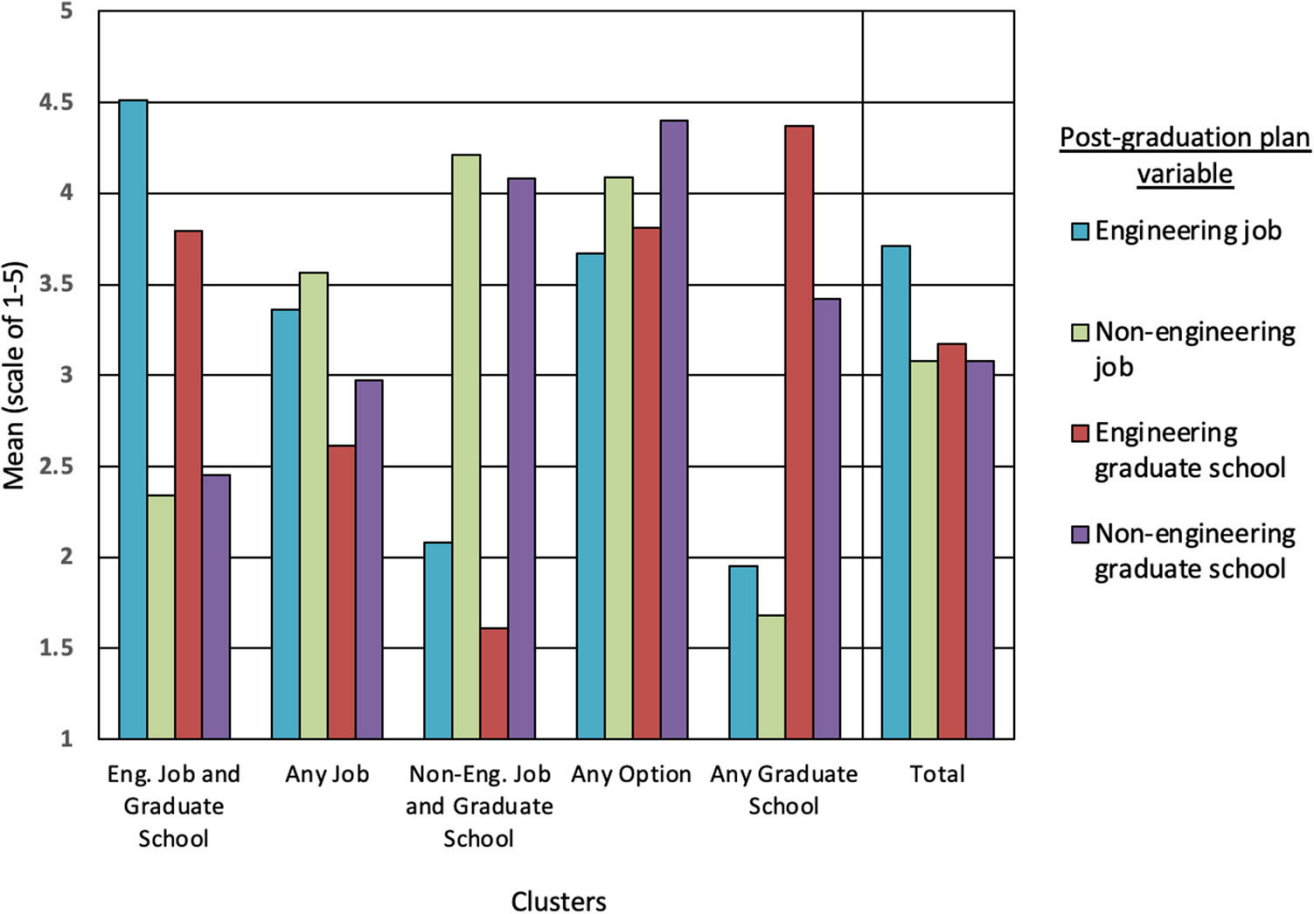
Column graph of clusters by post-graduation plan variable. *Y*-axis is the mean of the post-graduation plan variable. *X*-axis are the labels for the clusters; total indicates the average from the total sample. To the right of the chart is the legend.

Subsequently, the groupings were characteristically named by tabulating the means of each of the intended post-graduation plans by cluster. The reader is reminded that the scale for these variables is “1” definitely not, “2” probably not, “3” not sure, “4” probably yes, and “5” definitely yes. Specifically, responses greater than 3 are leaning towards an affirmative inclination for a particular post-graduation plan (work in an engineering job, work in a non-engineering job, go to graduate school in engineering, and go to graduate school outside of engineering*)*. Thus, the five clusters were named (from largest to smallest) as follows: *Engineering Job and Graduate School, Any Job, Non-Engineering Job and Graduate School, Any Option,* and *Any Graduate School.* Figure 2 illustrates these results. Table 5 lists the means and standard deviations of the clustering variables, and the size of the clusters.

**Table 5:** Mean and standard deviation of intended post-graduation plans by cluster.

	Eng. job and graduate school	Any job	Non-eng. job and graduate school	Any option	Any grad. school	Total
	*n* = 324	*n* = 228	*n* = 87	*n* = 58	*n* = 19	*n* = 716
	45.3%	31.8%	12.2%	8.1%	2.7%	100%
Engineering job	4.51 (0.58)	3.36 (0.86)	2.08 (0.89)	3.67 (0.80)	1.95 (0.85)	3.71 (1.13)
Non-engineering job	2.34 (0.74)	3.56 (0.64)	4.21 (1.18)	4.09 (0.66)	1.68 (0.75)	3.08 (1.10)
Engineering graduate school	3.79 (0.95)	2.61 (0.89)	1.61 (0.75)	3.81 (0.85)	4.37 (0.83)	3.17 (1.20)
Non-engineering graduate school	2.45 (0.99)	2.97 (1.02)	4.08 (0.81)	4.40 (0.89)	3.42 (1.12)	3.08 (1.23)
% Male	48.5	56.1	52.9	55.2	52.6	52.1
% Female	51.5	43.9	47.1	44.8	47.4	47.9

Mean (SD) for intended post-graduation plans are based on a scale of 1 to 5

#### Gender Representation by Cluster

To address research question 2, we examined gender representation (Table 5) within clusters. The results indicated that within each cluster, there was a statistically equivalent gender representation to the sample mean. Further, between clusters, there is not a statistically significant difference in the representation of women versus men (*F*(4,711) = 0.86, *p* = 0.486).

#### Engineering Attitudes by Cluster

Table 6 shows the student ratings on each of the attitudes by cluster. Means that share a letter in the superscript are statistically different using a Benjamini–Hochberg correction which controls for Type-I error (false positive) while also not over-inflating Type-II error (false negative). This is a sequential method of controlling for false discovery rate in multiple comparisons and yields greater power than the Bonferroni correction.^37^

**Table 6:** Mean and standard deviation of engineering attitude variables by cluster.

	Eng. job and graduate school	Any job	Non-eng. job and graduate school	Any option	Any grad. school	Total
	*n* = 324	*n* = 228	*n* = 87	*n* = 58	*n* = 19	*n* = 716
	45.3%	31.8%	12.2%	8.1%	2.7%	100%
Engineering academic self-efficacy	4.00 (0.67)	3.91 (0.65)	3.87 (0.80)	4.07 (0.64)	4.02 (0.67)	3.96 (0.68)
Engineering interest	4.48^ab^ (0.61)	4.20^ac^ (0.69)	4.08^bd^ (0.81)	4.45^cd^ (0.63)	4.42 (0.58)	4.34 (0.69)
Engineering utility value	4.50^ef^ (0.50)	4.21^egh^ (0.61)	3.94^fgij^ (0.70)	4.33^hi^ (0.69)	4.42^j^ (0.69)	4.18 (0.78)
Engineering attainment value	4.59^kl^ (0.53)	4.29^kmn^ (0.68)	4.01^lnop^ (0.77)	4.44^o^ (0.60)	4.66^pm^ (0.55)	4.41 (0.65)
Engineering professional identity^+^	5.20^qr^ (1.20)	4.68^qs^ (1.26)	4.68^rt^ (1.20)	5.33^st^ (1.16)	5.16 (1.63)	4.98 (1.26)

Mean (SD) student attitudes are based on a scale of 1 to 5

^+^Engineering professional identity is on a scale of 1 to 8

The table is to be examined by rows. Means that share a letter in the superscript are statistically different with Benjamini–Hochberg correction (largest adjusted *p* value < 0.03)

**Figure 3:**
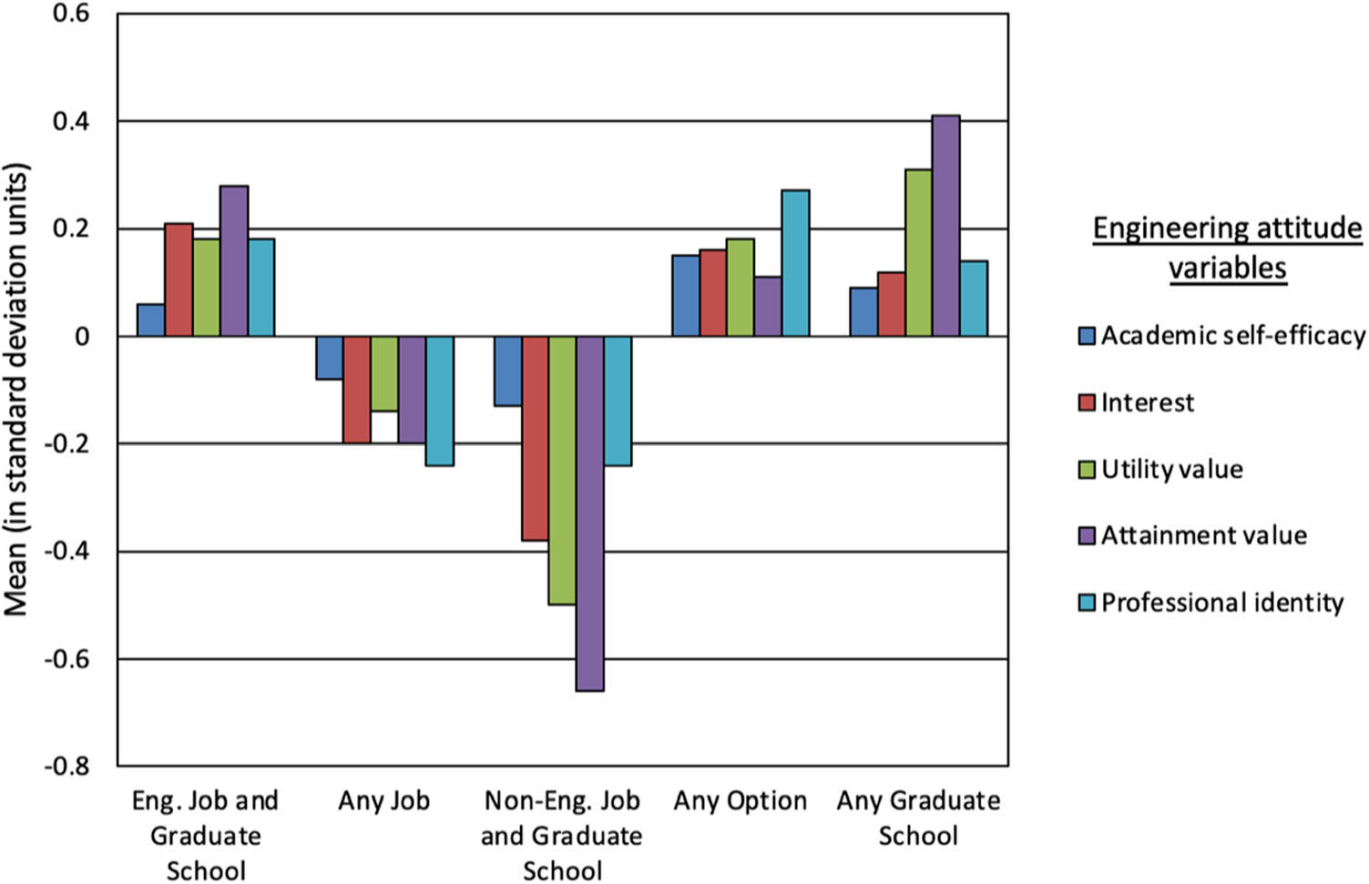
Column graph of engineering attitudes by cluster. *Y*-axis is the mean of the engineering attitude variables in standard deviation (SD) units. *X*-axis are the clusters variables. To the right of the chart is the legend.

For direct comparison, the ratings were standardized (i.e., recoded to have a mean of 0 and standard deviation of 1) and plotted in Fig. 3. Overall, the ratings for *Non-Engineering Job and Graduate School* and *Any Job* were below the standardized sample mean (0.00) whereas the remaining clusters– *Engineering Job and Graduate School, Any Option,* and *Any Graduate School–* were above the sample mean. When examining differences in engineering attitudes between clusters, the results revealed that there were no significant differences in engineering academic self-efficacy (*F*(4,771) = 1.30, *p* = 0.269). However, there were differences in the other attitudes between clusters: engineering interest (*F*(4,711) = 9.76, *p* = 0.000), engineering utility value (*F*(4,711) = 10.73, *p* = 0.000), engineering attainment value (*F*(4,711) = 19.08, *p* = 0.000), and engineering professional identity (*F*(4,711) = 8.57, *p* = 0.000).

Pairwise comparisons revealed several differences between clusters. Students in the *Engineering Job and Graduate School* cluster were higher than students in the *Any Job* and *Non-Engineering Job and Graduate School* clusters on engineering interest, engineering utility value, engineering attainment value and engineering professional identity. The differences in means between *Engineering Job and Graduate School* and *Any Job* were small–ranging from 0.32 to 0.47 (*p* < 0.001). Similarly, differences in means between *Engineering Job and Graduate School* and *Non-Engineering Job and Graduate School* were small to moderate, ranging from 0.41 to 0.68 (*p* < 0.001), for these same four attitudes with the exception of the difference in attainment value, which was large at 0.89 standard deviations (*p* < 0.001).

Additionally, students in the *Any Option* cluster were higher than *Non-Engineering Job and Graduate School* on engineering interest, engineering utility value, engineering attainment value and engineering professional identity. Differences in means between *Any Option* and *Non-Engineering Job and Graduate School* were moderate, ranging from 0.51 to 0.69 standard deviations (*p* < 0.01). *Any Option* was also significantly higher than *Any Job* on engineering interest, engineering utility value and engineering professional identity. The differences in means between *Any Option* and *Any Job* were small to moderate–ranging from 0.33 to 0.52 standard deviations (*p* < 0.05).

Further, *Any Graduate School* was significantly higher than *Non-Engineering Job and Graduate School* on utility value and attainment value. Differences in means for these two variables were both large at 0.81 and 1.00 standard deviations, respectively (*p* < 0.01). There was a moderate difference in attainment value between *Any Graduate School* and *Any Job* (difference = 0.58; *p* < 0.05). Lastly, *Any Job* was higher than *Non-Engineering Job and Graduate School* on utility value and attainment value. Differences in means were small at 0.36 and 0.42 standard deviations, respectively (*p* < 0.01).

In sum, the most frequent and largest differences between clusters were found on engineering utility value and engineering attainment value. The largest differences were found between the *Any Graduate School* and *Non-Engineering Job and Graduate School* clusters for both variables and *Engineering Job and Graduate School* and *Non-Engineering Job and Graduate School* for only attainment value. Secondly, students in the *Engineering Job and Graduate School* and *Any Option* clusters were higher than those in the *Any Job* and *Non-Engineering Job and Graduate School* clusters on interest, utility value, and professional identity. The reader is reminded that there were no statistically significant differences in engineering academic self-efficacy between clusters.

### Gender Differences Within Clusters

To answer research question 3, we conducted t-tests on the differences in means for male and female students in each cluster on the engineering attitude variables. As seen in Table 7, we performed t-tests with a Benjamini-Hochberg correction to adjust for the multiple comparisons (with adjusted thresholds for statistical significance between *p* < 0.01 and *p* < 0.03 for the smallest and largest value respectively). Using this criterion, although the means for male students are often higher than those for female students on many of the attitudinal variables, few differences reach statistical significance. For three clusters (*Engineering Job and Graduate School*, *Any Job,* and *Any Option)*, there were significant gender differences in self-efficacy, such that female students report significantly lower ratings than their male peers (*p* < 0.01). Specifically, among students in the *Engineering Job and Graduate School* cluster, the gender difference in average self-efficacy (i.e., effect size) was moderate in size, at 0.46 standard deviations. For those students in the *Any Job* cluster, the gender difference was large and nearly 1 standard deviation (*d* = 0.94). The standardized difference in the means for those in the *Any Option* cluster was also large (*d* = 0.71).

**Table 7:** Means and standard deviations of engineering attitude variables by gender.

	Eng. job and graduate school		Any job		Non-eng. job and graduate school		Any option		Any graduate school	
	Male	Female	Male	Female	Male	Female	Male	Female	Male	Female
	*n* = 157	*n* = 167	*n* = 128	*n* = 100	*n* = 46	*n* = 41	*n* = 32	*n* = 26	*n* = 10	*n* = 9
Engineering academic self-efficacy	4.16^a^ (0.71)	3.85^a^ (0.60)	4.16^b^ (0.55)	3.60^b^ (0.65)	4.06 (0.75)	3.66 (0.82)	4.26^c^ (0.63)	3.83^c^ (0.58)	4.10 (0.67)	3.93 (0.69)
Engineering interest	4.50 (0.64)	4.46 (0.59)	4.30^d^ (0.69)	4.08^d^ (0.68)	4.25 (0.79)	3.89 (0.79)	4.50 (0.60)	4.38 (0.67)	4.50 (0.53)	4.33 (0.66)
Engineering utility value	4.31 (0.69)	4.34 (0.72)	4.10 (0.79)	4.03 (0.78)	3.96 (0.82)	3.61 (0.97)	4.41 (0.80)	4.23 (0.51)	4.70 (0.48)	4.11 (0.78)
Engineering attainment value	4.55 (0.53)	4.63 (0.51)	4.28 (0.67)	4.30 (0.70)	3.96 (0.74)	4.07 (0.81)	4.33 (0.66)	4.58 (0.50)	4.85 (0.24)	4.44 (0.73)
Engineering professional identity^+^	5.37^e^ (1.29)	5.04^e^ (1.08)	4.85^f^ (1.23)	4.47^f^ (1.28)	4.78 (1.40)	4.57 (0.93)	5.42 (1.29)	5.21 (0.99)	5.35 (1.23)	4.94 (2.05)

Mean (SD) student attitudes are based on a scale of 1 to 5

The comparison of the means are between male and female within the same cluster. Means that share a letter in the superscript are statistically different with Benjamini-Hochberg correction (largest adjusted *p* value < 0.03)

^+^Engineering professional identity is on a scale of 1–8

There were few differences between clusters in the remaining four variables. The only significant gender difference in engineering interest was for students in the *Any Job* cluster (*p* = 0.018; *d* = 0.32). Further, there was a significant gender difference in engineering professional identity, for those in the *Engineering Job and Graduate School* cluster (*p* = 0.014) and *Any Job* cluster (*p* = 0.025) such that female students reported lower ratings than their male peers. However, the effect sizes for both these differences were small at 0.28 and 0.30 standard deviations, respectively. It should be noted there were no significant gender differences for attainment value or utility value. There were no gender differences in attitudes for students in the *Non-Engineering Job and Graduate School* or *Any Graduate School* clusters. The latter result is likely due to the small number of males (*n* = 10) and females (*n* = 9) which diminishes statistical power.

## Discussion

In response to research question 1, the results did not find support for a cluster solution that would have produced a binary ‘stay versus leave’ categorization as typically used in research on engineering persistence. Rather, the results revealed BME students form five clusters of intended post-graduation plans: *Engineering Job and Graduate School, Any Job, Non-Engineering Job and Graduate School, Any Option,* and *Any Graduate School* (Table 5). Only two of the five clusters encapsulated discrete options (i.e., *Engineering Job and Graduate School* and *Non-Engineering Job and Graduate School*). Three other clusters, composing the remaining 43% of the sample, captured overlapping engineering and non-engineering plans. Both the *Any Job* and *Any Graduate School* clusters reflect an affinity for pursuing plans based on a certain career type; students in these clusters are simultaneously considering engineering and non-engineering options. The *Any Option* cluster was identified as having an inclination for post-graduation plans in all four categories.

Given the way that many prior studies have framed persistence, it is important to acknowledge the existence of a *Non-Engineering Job and Graduate School* cluster of students who are persisting in engineering degrees but are not particularly interested in an engineering career or engineering graduate school. While one motivation for this work was driven by the assumption that biomedical engineering students are considering a graduate school pathway towards medical or other professional schools, we note that only 11% of students collectively comprised the *Any Graduate School* and *Any Option* clusters which include this type of future plan. The reality may be more complex and worthy of future study.

In response to research question 2, men and women were equally represented across clusters, indicating neither gender was more likely to prefer certain career plans over another (Table 5). This is somewhat consistent with other work on gender differences in career outcomes among BME students. For example, Ortiz-Rosario *et al.*^25^ found no gender difference in the intention to pursue further education—which included graduate and professional school. Yet in the same study, the authors found a larger proportion of women intending to pursue an industry placement after graduation as compared to men, who were still looking for employment. More research on gender differences in intended and actual career choice for BME students is warranted.

While gender did not differentiate who was in each cluster, engineering attitudes did. Specifically, with the exception of self-efficacy, there were notable differences between the clusters in all of the other engineering attitudes studied (engineering interest, utility value, attainment value, and professional identity). Overall, there was a clear separation in which those in the *Engineering Job and Graduate School, Any Option,* and *Any Graduate School* clusters reported means above the total sample average, and those in the *Non-Engineering Job and Graduate School* and *Any Job* clusters reported means below the mean for each of the engineering attitudes (Fig. 2). *Engineering Job and Graduate School* had consistently more positive attitudes than *Non-Engineering Job and Graduate School* and *Any Job,* but was not significantly higher than *Any Graduate School* or *Any Option.* In fact, students in the *Any Option* cluster (i.e., those open to all options), like those in the *Engineering Job and Graduate School* cluster, also had consistently higher attitudes than *Non-Engineering Job and Graduate School.* (The same result was expected for *Any Graduate School* yet significant differences were not found; this was likely due to the small size of this cluster (*n* = 19)).

It is understandable that students in the *Engineering Job and Any Graduate School* cluster would have the highest engineering attitudes. This would suggest an alignment of pro-engineering attitudes with intended career plans in engineering–a logical conclusion. However, it is less clear why those in two other clusters, *Any Graduate School* or *Any Option,* would report equally high means. These were the smallest of the clusters identified, and supplemental analyses (not included here due to space limitations) revealed that they also had the highest proportions of lower division students. Thus, these high ratings may be an effect of comparatively younger and/or less career-informed students with overly optimistic engineering attitudes. As new-comers to engineering, younger students have not had the opportunity to take as many engineering courses or engage more meaningly in the engineering practices such as work-related internships, research or cooperative experiences. Thus, we infer their experiential knowledge of engineering as a profession is limited as compared to older students. Future work would need to be conducted to investigate these assumptions.

Findings further revealed students in the *Non-Engineering Job and Graduate School* cluster appear to report the lowest engineering attitudes for each attitude except engineering professional identity, which would logically suggest that students in this cluster have overall lower attitudes as associated with motivation to continue pursuing engineering. Yet statistical differences were only found among two attitude variables; students in this cluster were statistically significantly lower than students in every other cluster on only engineering utility value and attainment value. However, it is noteworthy that at the same time, students in this cluster had statistically similar levels of self-efficacy, interest and identity to those in the *Any Job* cluster. Thus, students that report wanting to pursue only non-engineering options did not have universally the lowest attitudes as evidenced by the statistical comparisons between clusters.

Additionally, an intriguing finding was observed regarding students in the *Any Job* cluster. Students in this cluster, those with an inclination for either an engineering or non-engineering job, consistently had attitudes that were below the mean. Yet, *Any Option* and *Any Graduate School* (also clusters with engineering and non-engineering inclinations) showed engineering attitudes above the mean, and subsequently, those in the *Any Job* cluster were found to have significantly lower interest, utility value and identity compared to these other two clusters. Thus, despite these three clusters being comprised of students reporting a mix of engineering and non-engineering inclinations, there were significant distinctions in their attitudinal profiles. Further research can investigate the reasoning for these differences. One plausible explanation is that those in the *Any Job* cluster may be somewhat indifferent to engineering as a discipline, and their initial motivations for studying engineering in the first place were perhaps related to ideas about being able to later secure a high-paying job.

Beyond the fact that attitudes differed across clusters, regarding research question 3, we also found evidence of gender differences in attitudes within some clusters (Table 7). Specifically, within the *Engineering Job and Graduate School, Any Job* and *Any Option* clusters, there were significantly lower mean self-efficacy ratings for female students compared to male students. Further, women reported significantly lower engineering professional identity than men in the *Engineering Job and Graduate School* and *Any Job* clusters. This is consistent with research which finds a persistent gender gap in self-efficacy for women across STEM^10^ and in identification with engineering specifically.^23^ Yet at the same time, it is somewhat surprising to find evidence of these gaps within a nearly gender-equitable discipline. Further, the only gender difference in engineering interest was found for students in the *Any Job* cluster. Among the other two engineering attitudes (attainment value and utility value), there were no statistically significant gender differences within clusters. Therefore, among men and women with the same intended career plans, both genders report similar levels of wanting to succeed in engineering and perceived usefulness of engineering. Thus, while some attitudes (self-efficacy, interest and identity) were gender-differentiated, other attitudes (utility value and attainment value) were not. Our results point to the complexity of understanding gender differences in engineering; by considering gendered patterns simultaneously across an array of engineering attitudes we are able to pinpoint where men and women are similar and where they diverge.

## Limitations

As with any study, there are limitations. First, the data were collected at a single institution, and thus the findings may not be representative of biomedical engineering programs at other institutions. Regarding the measures asking students for their post-graduation plans, the questions are worded as “engineering” or “non-engineering” and do not make the distinction between engineering and engineering-related choices such as medical sciences. Additionally, non-engineering graduate school is inclusive of all further graduate education including professional schools such as medical, law or business school, and master’s degrees were not distinguished from doctoral degrees in either of the graduate school options. Lastly, due to a small sample size, the data could not be disaggregated further by race/ethnicity.

## Conclusion and Implications

In conclusion, this study has several implications for BME departments. Biomedical engineering students should not be presumed to only want to pursue engineering careers post-graduation nor hold unilaterally high attitudes related to engineering. As evidenced, students show variation in both their intended post-graduation plans and attitudes towards engineering. Students may need more help understanding different career options, rather than simply being offered resources aligned with different career tracks. Individualized experiences such as internships and undergraduate research help a select group of students gain access to mentors and role models; however, BME curricula should also be examined with regard to its impact on student experiences. For example, coursework assignments could span different industries and settings, with explanation of how they relate to professional options. Future work would need to be conducted to investigate these assumptions in the BME context as there is extensive research that career planning courses support career exploration and engagement for undergraduates students in higher education^29^ including a few studies of STEM majors.^3^,^4^,^28^ Given the multiple career paths in BME, programs could structure courses to include opportunities for students to discuss how different content and skills apply and are useful in a variety of careers. This is particularly important considering the already documented discrepancy between BME curricula and stakeholder expectations of BME graduates.^30^

Thus, while BME does require similar programs of study to some other disciplines such as electrical engineering, the post-graduation pathways of biomedical engineering graduates are vast and various.^18^ More effectively providing information about these pathways to students across all stages (even before college) can potentially recruit and retain a swath of students that might not otherwise consider entering or persisting in engineering if they do not see a pathway forward in the major. Beneficial to all, more explicit discussions of careers in and out of engineering can help individuals make the transition into the workforce—graduate student, professional or otherwise.

Lastly, we want to emphasize that the effectiveness of these aforementioned implications partially depends on larger collective efforts to promote gender equity. Given the systemic influences on students’ intended career choices proposed by EVT,^13^ it would be appropriate to consider whether interventions can be employed to boost female students’ self-efficacy and identification with engineering. For example, continuing to transform minds in regards to stereotypes about gender-appropriate careers can potentially address some of the biases that occur at much earlier stages in students’ educational pathways both inside and outside of school. Additionally, helping both women and men to confront their own gender-biased beliefs could also lead to decreasing discrepancies in self-efficacy. In fact, women’s lower ratings of their self-efficacy could be relative to men’s over-inflation of perceptions of self-confidence. And although this study examined academic self-efficacy and not professional self-efficacy^9^ (their confidence to succeed or perform in the career setting), gender differences in self-efficacy that persist after entry into the workforce may be a reason that women choose to leave STEM fields, in conjunction with the other issues including harassment and discrimination that women experience in the workforce.

Yet at the same time, individual agency should still be acknowledged in that students’ decisions to pursue non-STEM careers after graduation may be a choice to leave rather than being forced out of STEM career fields due to lack of opportunity or ability. Future work should replicate and expand this research in other BME departments to determine if these findings are truly generalizable as related to the null findings of gender representation with clusters and the significant results of gender differences in attitudes within clusters. Coupled with qualitative methods to understand students’ career decision-making processes, such as interviews and focus groups, researchers can more deeply probe social-psychological and contextual factors such as gender socialization, gender stereotypes, and the origin of attitudinal beliefs. Mixed methods research designs are especially important for researchers that employ cluster analysis in which there is the possibility of discovering small sub-populations in data that are too small for further investigation with quantitative methodologies. Studies spanning across the K-16 engineering pathway and extending past degree attainment into industry, academia, and other career settings would further illuminate the understanding of discipline-specific career decision-making as individuals negotiate their future choices based on past and present experiences, personal and social identities, attitudes and beliefs, and conceptions of their future selves.
